# *Propionibacterium acnes* Accelerates Intervertebral Disc Degeneration by Inducing Pyroptosis of Nucleus Pulposus Cells via the ROS-NLRP3 Pathway

**DOI:** 10.1155/2021/4657014

**Published:** 2021-02-01

**Authors:** Guoqing Tang, Xiaoguang Han, Zhijie Lin, Hongbin Qian, Bing Chen, Chengliang Zhou, Yong Chen, Weimin Jiang

**Affiliations:** ^1^Orthopedic Center, The First Affiliated Hospital of Soochow University, Suzhou 215000, China; ^2^Kunshan Hospital of Traditional Chinese Medicine, Kunshan 215300, China; ^3^Institute of Translational Medicine, Medical College, Yangzhou University, Yangzhou 225001, China; ^4^Department of Spine Surgery, Beijing Jishuitan Hospital, Beijing 100035, China; ^5^Jiangsu Key Laboratory of Experimental & Translational Non-coding RNA Research, Yangzhou 225001, China

## Abstract

Our previous study verified the occurrence of *Propionibacterium acnes* (*P*. *acnes*), a low-virulence anaerobic bacterium, latently residing in degenerated intervertebral discs (IVDs), and the infection had a strong association with IVD degeneration. We explored whether *P*. *acnes* induces nucleus pulposus cell (NPC) pyroptosis, a more dangerous cell death process than apoptosis, and accelerates IVD degeneration via the pyroptotic products interleukin- (IL-) 1*β* and IL-18. After coculturing with *P*. *acnes*, human NPCs showed significant upregulation of NOD-like receptor 3 (NLRP3), cleaved IL-1*β*, cleaved caspase-1, and cleaved gasdermin D in response to the overexpression of IL-1*β* and IL-18 in a time- and dose-dependent manner. In addition, the gene expression of inflammatory factors and catabolic enzymes significantly increased in normal NPCs when cocultured with pyroptotic NPCs in a transwell system, and the adverse effects were inhibited when NPC pyroptosis was suppressed. Furthermore, inoculation of *P*. *acnes* into the IVDs of rats caused significant pyroptosis of NPCs and remarkable IVD degeneration. Finally, coculture of NPCs with *P*. *acnes* induced the overexpression of reactive oxygen species (ROS) and NLRP3, while inhibition of both factors reduced NPC pyroptosis. Therefore, *P*. *acnes* induces NPC pyroptosis via the ROS-NLRP3 signaling pathway, and the pyroptotic NPCs cause an IVD degeneration cascade. Targeting the *P*. *acnes*-induced pyroptosis of NPCs may become an alternative treatment strategy for IVD degeneration in the future.

## 1. Introduction

Intervertebral disc (IVD) degeneration is a serious public health problem. A variety of clinical symptoms are caused by IVD degeneration, such as sciatica, lower back pain, and physical dysfunction, which significantly reduce the quality of life and work productivity of patients and increase the burden on the medical system [1]. Unfortunately, the exact mechanism of IVD degeneration is still unclear and treatment is limited.

There have been several hypothetical explanations for IVD degeneration: excessive axial or shear force, biochemical factors, gene diversity, and low-virulence anaerobic bacterial infection [1]. Since the first report by Stirling et al. in 2001 [2], the pooled bacterial infection rate of IVDs over the past decade has been 25.3% [3]. Of the various infecting low-virulence anaerobic bacteria, *Propionibacterium acnes* is the most important for IVD degeneration, with an IVD infection rate of around 15.5% [3]. Our previous study reported the infection rate of *P*. *acnes* to be 26.25% within degenerated IVDs, and this bacterial infection has a strong clinical association with IVD herniation in younger patients [4, 5].

Accumulated evidence indicates that *P*. *acnes* infection has a strong relationship with IVD degeneration [6], Modic changes [7], and lower back pain [8] without causing discitis and, therefore, differs from *Staphylococcus aureus* infection [9]. For example, clinical evidence revealed that *P*. *acnes*-infected IVDs show more severe degeneration than uninfected IVDs [10]. Inoculation of *P*. *acnes* into the IVDs of rabbits and rodents resulted in obvious IVD degeneration, but symptoms of discitis did not show on radiological and histological analyses [9, 11]. Thus, the identification of *P*. *acnes* as the etiology for IVD degeneration may provide a new treatment strategy in the future.

One of the pathophysiological processes in IVD degeneration is the programmed cell death of nucleus pulposus cells (NPCs), as NPCs are critical to the maintenance of the mechanical and biochemical homeostases of IVDs [12]. It was initially thought that apoptosis is the main death form of NPCs, and previous research demonstrated that *P*. *acnes* causes the apoptosis of NPCs, which induces IVD degeneration [13]. However, recent studies revealed that when tissue is infected by bacteria, the cells form inflammasomes and undergo death in the form of pyroptosis [14, 15]. This is more dangerous for tissue than apoptosis because pyroptotic cells release a lot of interleukin- (IL) 1*β* and IL-18, leading to further inflammatory damage [15]. Therefore, pyroptosis has also been called “inflammatory programmed cell death” [15].

A previous study showed that *P*. *acnes* can cause the pyroptosis of a monocytic leukemia cell line (THP-1) [16]. Hence, we wanted to know whether *P*. *acnes* can cause the pyroptosis of NPCs and the subsequent IVD degeneration cascade. Furthermore, the mechanism of how *P*. *acnes* induces the pyroptosis of NPCs was also explored. This study is aimed at investigating the relationship between *P*. *acnes* infection and NPC pyroptosis, and our findings may provide insights into new methods of preventing and treating IVD degeneration.

## 2. Materials and Methods

### 2.1. Inoculation of *P*. *acnes* into Caudal Intervertebral Discs of Rats

Male Sprague-Dawley rats weighing around 250-300 g were used for an *in vivo* study. All animal experiments were performed in accordance with a protocol approved by the Institute of the Animal Care and Use Committee of Soochow University and the National Institutes of Health guide for the care and use of laboratory animals (NIH Publications No. 8023, revised 1978). For anesthetization, 2.5% sodium pentobarbital was used via intraperitoneal injection, and the caudal (Ca) vertebrae Ca6/7 to Ca8/9 were identified as the target IVDs. After penetration to a depth of approximately 2.0-2.5 mm, 2.5 *μ*l of *P*. *acnes* (optical density of 600 nm (OD600) = 3.0) or saline was inoculated into the IVDs using a microsyringe with a 28-gauge needle (Hamilton, Nevada, USA). To prevent contamination, all instruments and operations were performed under sterile conditions.

### 2.2. Preparation of *P*. *acnes* Inoculum

For the *in vivo* and *in vitro* experiments, a standard strain of *P*. *acnes* (ATCC: 6919, GIM: 1.243, purchased from Guangdong Microbiology Culture Center, Guangdong, China) was cultured in Gifu Anaerobic Medium (GAM) broth (Nissui, Tokyo, Japan) for 3 d at 37°C under anaerobic conditions. Then, the bacteria were harvested and suspended in 1x phosphate-buffered saline (PBS) for the next experiment.

### 2.3. Cocultures of NPCs and *P*. *acnes*

Human nucleus pulposus tissues were obtained from four patients (two males and two females with a mean age of 31.5 years) with IVD herniation, and the cells were cultured. Cell samples from different patients were kept separate from each other. All experiments were carried out in triplicate, and NPCs from humans were cultured in passages 2 to 3.

For the coculture, the bacteria were harvested from 3-day cultures at the stationary phase and washed twice with PBS. The bacterial density was adjusted to an OD600, and *P*. *acnes* were added to the cell culture (5 × 10^5^ cells/well) in a 6-well culture plate at a 100 : 1, 10 : 1, and 1 : 1 multiplicity of infection (MOI) without antibiotics. After 0, 1, 2, and 4 h, the cocultured cells were washed three times with PBS and prepared for late-stage experiments. For the transwell coculture (pore size of the membrane: 0.4 *μ*m, Corning Transwell®, Corning Inc., NY, USA), the NPCs were plated on inserts, and the inserts were placed in 12-well plates containing adherent NPCs and *P*. *acnes* for 12 h.

### 2.4. Western Blotting Analysis

In the western blotting analysis, the samples were incubated with the inflammasome antibody sampler kit (cat. no. 32961T, CST Inc., MA, USA), anti-human gasdermin D (cat. no. 96458S, CST Inc., MA, USA), anti-rat IL-1*β* (cat. no. 500-P80-100, PeproTech, NJ, USA), anti-rat IL-18 (cat. no. ab191860, Abcam, UK), anti-rat caspase-1 (cat. no. ab1872, Abcam, UK), anti-rat collagen II (cat. no. ab34712, Abcam, UK), and anti-rat aggrecan (cat. no. ab36861, Abcam, UK) overnight, then incubated with horseradish peroxidase-conjugated secondary antibody, goat anti-rabbit IgG (1 : 2,000 dilution; cat. no. CW0103s; CW Bio, Beijing, China), or goat anti-mouse IgG (1 : 2,000 dilution; cat. no. CW0102s; CW Bio, Beijing, China) at room temperature for 2 h. GAPDH and *β*-actin were used as the internal controls. The bands were visualized using chemiluminescence (Pierce Biotechnology Inc., IL, USA) and analyzed using Fusion FX7 (Vilber Lourmat, Marne-la-Vallée, France).

### 2.5. Real-Time Quantitative PCR

Total RNA was isolated using the TRIzol reagent (Invitrogen, Life Technologies Corp., CA, USA), and cDNA was synthesized from 1 *μ*g of total RNA using reverse transcriptase (Takara, Shiga, Japan). An ABI 7500 Sequencing Detection System (Applied Biosystems, CA, USA) was used for qRT-PCR with the SYBR Premix Ex Taq Kit (Takara, Shiga, Japan). Cycling conditions were as follows: 40 cycles of denaturation at 95°C for 5 s and amplification at 60°C for 24 s. The housekeeping gene GAPDH served as a control, and all reactions were run in triplicate. The primer sequences (Sangon Biotech, Shanghai, China) used in this study were as follows: human tumor necrosis factor-*α* (TNF-*α*): forward 5′-TCGTAGCAAACCACCAAGCA-3′, reverse 5′-TCGTAGCAAACCACCAAGCA-3′; human interleukin-1*β* (IL-1*β*): forward 5′-GCACAGTTCCCCAACTGGTA-3′, reverse 5′-GGAGACTGCCCATTCTCGAC-3′; human interleukin-6 (IL-6): forward 5′-CATTCTGTCTCGAGCCCACC-3′, reverse 5′-AGTCTCCTCTCCGGACTTGT-3′; human matrix metallopeptidase 3 (MMP3): forward 5′-TTTGGCCGTCTCTTCCATCC-3′, reverse 5′-GCATCGATCTTCTGGACGGT-3′; human matrix metallopeptidase 13 (MMP13): forward 5′-ACCATCCTGTGACTCTTGCG-3′, reverse 5′-TTCACCCACATCAGGCACTC-3′; human A disintegrin and metalloproteinase with thrombospondin motifs 4 (ADAMTS4): forward 5′-ACCGATTACCAGCCTTTGGG-3′, reverse 5′-CCGACTCCGGATCTCCATTG-3′; human A disintegrin and metalloproteinase with thrombospondin motifs 5 (ADAMTS5): forward 5′-CCGAACGAGTTTACGGGGAT-3′, reverse 5′-TGTGCGTCGCCTAGAACTAC-3′; and human *β*-actin: forward 5′-AACCTTCTTGCAGCTCCTCCG-3′, reverse 5′-CCATACCCACCATCACACCCT-3′. Target gene expression levels were normalized to the expression of *β*-actin using the 2^-*ΔΔ*^Ct method. All data were then normalized to the average of the control group.

### 2.6. Enzyme-Linked Immunosorbent Assay

Supernatants of NPC cultures were harvested after centrifugation at 10,000 × *g* and 4°C for 5 min. The quantification of IL-1*β* and IL-18 was determined according to the specific human IL-1*β* ELISA kit (cat. no. DY201-05, R&D systems, MN, USA) and human IL-18 ELISA kit (cat. no. DY318-05, R&D systems, MN, USA), respectively, based on the manufacturer's instructions.

### 2.7. Histological Examination

For histological observation, the rat IVDs were initially fixed in 4% formaldehyde for 24 h. Routine paraffin embedding was performed for all samples, which were then sectioned to 5 *μ*m. Routine H&E, Safranin O, and Alcian Blue staining were conducted following the manufacturer's instructions. The stained samples were observed and photographed under a microscope (Axio, Carl Zeiss, Oberkochen, Germany).

### 2.8. Flow Cytometry of DCFH-DA and FAM FLICA Caspase-1

The ROS in the NPCs were quantified with dichloro-dihydro-fluorescein diacetate (DCFH-DA, 10 *μ*M, S0033, Beyotime, Shanghai, China). After treatment with DCFH-DA (2 *μ*l of DCFH-DA dissolved in 2 ml RPMI 1640) at 37°C for 20 min, the fluorescence was analyzed using a FACSVerse (Becton Dickinson, Sunnyvale, CA, USA) flow cytometer with excitation at 488 nm and emission at 525 nm.

The proportion of pyroptosis in NPCs was measured with the FAM FLICA™ Caspase-1 Kit (ICT098, Bio-Rad, CA, USA). After incubation with FAM FLICA (10 *μ*l of 30x FLICA solution in 290 *μ*l RPMI 1640) at 37°C for 30 min, the pyroptotic NPCs were analyzed using flow cytometry with excitation at 494 nm and emission at 520 nm.

### 2.9. MRI Examination of Rodent IVDs

The samples were harvested and fixed in 4% formaldehyde for 24 h. Then, the samples were scanned with a coil of RF RES 400 1H 089/023 M.BR QSN TR in an MRI system (Bruker Biospec 94/20 USR, Bruker Corporation, MA, USA). The parameters were as follows: tr = 617.966 ms, te = 18 ms, average = 6, maxtr = 384∗256, fov = 32∗32 mm, slice thickness = 1 mm, and fat suppression. All MRI images were evaluated independently by two authors.

### 2.10. Statistics

Continuous variables were expressed as mean ± SD. For two groups, Student's *t*-test was used when the variables were normally distributed. For three groups, the one-way ANOVA test was conducted and followed by post hoc analysis of the Bonferroni test. Statistical significance was assumed at *P* < 0.05.

## 3. Results

### 3.1. *P*. *acnes* Induced the Pyroptosis of NPCs

**Figure 1:**
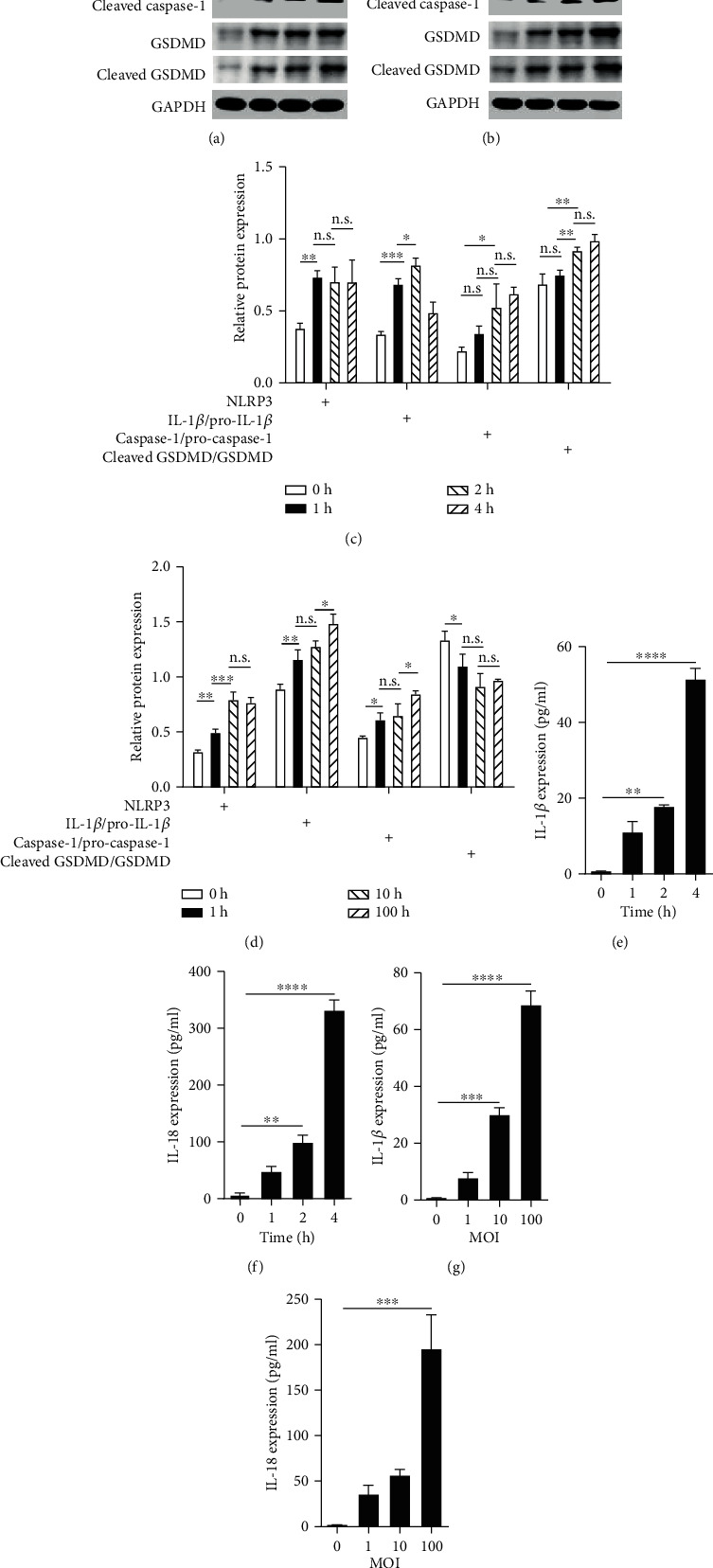
*P*. *acnes* induced the pyroptosis of NPCs. (a, b) NPCs cocultured with *P*. *acnes* for different times or at various concentrations resulted in a gradual increase in pyroptosis-related proteins, such as NLRP3, pro-IL-1*β*, cleaved IL-1*β*, pro-caspase-1, cleaved caspase-1, gasdermin D, and cleaved gasdermin D, in a time-dependent or dose-dependent manner. (c, d) On further analysis, the relative expression of NLRP3 and the ratios of cleaved IL-1*β* to pro-IL-1*β*, cleaved caspase-1 to pro-caspase-1, and cleaved gasdermin D to gasdermin D gradually increased. (e–h) On ELISA, the products of pyroptosis, IL-1*β* and IL-18, significantly increased in a time- or dose-dependent manner in the coculture supernatants. One-way ANOVA and Bonferroni tests were used for statistical analysis. ^∗^*P* < 0.05, ^∗∗^*P* < 0.01, and ^∗∗∗^*P* < 0.001 when comparisons were made between groups. All of the values were expressed as mean ± SD.

When *P*. *acnes* was cocultured with NPCs for different durations and MOI, the NPCs showed remarkable pyroptosis. As depicted in Figures 1(a)–1(d), the western blotting analysis showed that when cocultured for 0, 1, 2, or 4 h or at an MOI of 0, 1, 10, or 100, the expression of NLRP3 and the ratios of cleaved IL-1*β* to pro-IL-1*β*, cleaved caspase-1 to pro-caspase-1, and cleaved gasdermin D to full-length gasdermin D were gradually upregulated in a time- or dose-dependent manner. Additionally, the concentration of IL-1*β* and IL-18 in the supernatant also gradually increased in a time- or dose-dependent manner, as shown in Figures 1(e)–1(h). Therefore, it is reasonable to conclude that *P*. *acnes* was able to induce the pyroptosis of NPCs.

### 3.2. *P*. *acnes*-Induced Pyroptotic NPCs Resulted in an Overexpression Cascade of Inflammatory Factors and Catabolic Enzymes in Normal NPCs

**Figure 2:**
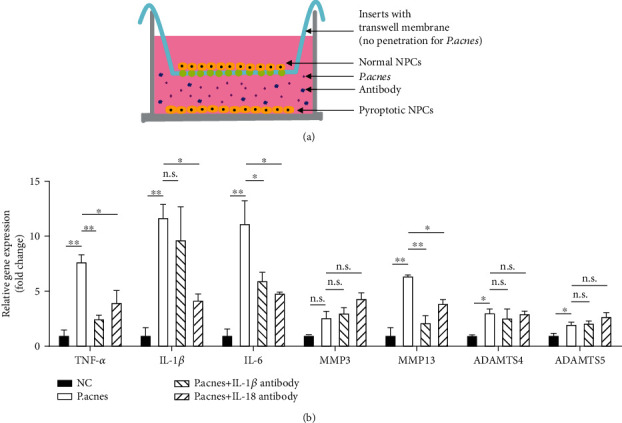
*P*. *acnes*-induced pyroptotic NPCs resulted in an overexpression cascade of inflammatory factors and catabolic enzymes in normal NPCs. (a) When *P*. *acnes*-induced pyroptotic NPCs were cocultured with normal NPCs in a transwell apparatus, the products of *P*. *acnes*-induced pyroptotic NPCs caused significant upregulation of TNF-*α*, IL-1*β*, IL-6, MMP3, MMP13, ADAMTS4, and ADAMTS5 gene expression. (b) When the products of pyroptosis were inhibited by antibodies to IL-1*β* or IL-18, expression of the previously upregulated genes significantly decreased, suggesting that the products of *P*. *acnes*-induced pyroptotic NPCs have devastating effects on normal surrounding NPCs. One-way ANOVA and Bonferroni tests were used for statistical analysis. ^∗^*P* < 0.05 and ^∗∗^*P* < 0.01 when comparisons were made between groups. All of the values were expressed as mean ± SD.

Distinct from apoptotic cells, pyroptotic cells produce large amounts of IL-1*β* and IL-18, both of which have catastrophic effects on the surrounding normal cells. To explore whether pyroptotic NPCs cause an overexpression cascade of inflammatory factors and catabolic enzymes in normal NPCs, we established a transwell cocultured system in which *P*. *acnes* only induced NPC pyroptosis in the bottom chamber, and the bacteria could not penetrate the transwell membrane to affect normal NPCs in the upper chamber (the pore size of the membrane from Corning Transwell® is 0.4 *μ*m, which is smaller than the average size of *P*. *acnes* at 1-2 *μ*m; see illustration in Figure 2(a)). Therefore, only the IL-1*β* or IL-18 products from the *P*. *acnes*-induced pyroptotic NPCs in the bottom chamber could pass through the transwell membrane and affect the normal NPCs.

As depicted in Figure 2(b), the normal NPCs had significantly increased TNF-*α*, IL-1*β*, IL-6, MMP13, ADAMTS4, and ADAMTS5 expression. In contrast, neutralization of IL-1*β* with a specific antibody significantly ameliorated the expression of TNF-*α*, IL-6, and MMP13, while neutralization of IL-18 with a specific antibody significantly attenuated the upregulation of TNF-*α*, IL-1*β*, IL-6, and MMP13. The results suggest that the pyroptotic NPCs induced the overexpression of inflammatory factors and catabolic enzymes, which surrounded the normal NPCs, via the pyroptotic products IL-1*β* and IL-18.

### 3.3. Inoculation of *P*. *acnes* Caused Pyroptosis of Nucleus Pulposus Cells and IVD Degeneration

**Figure 3:**
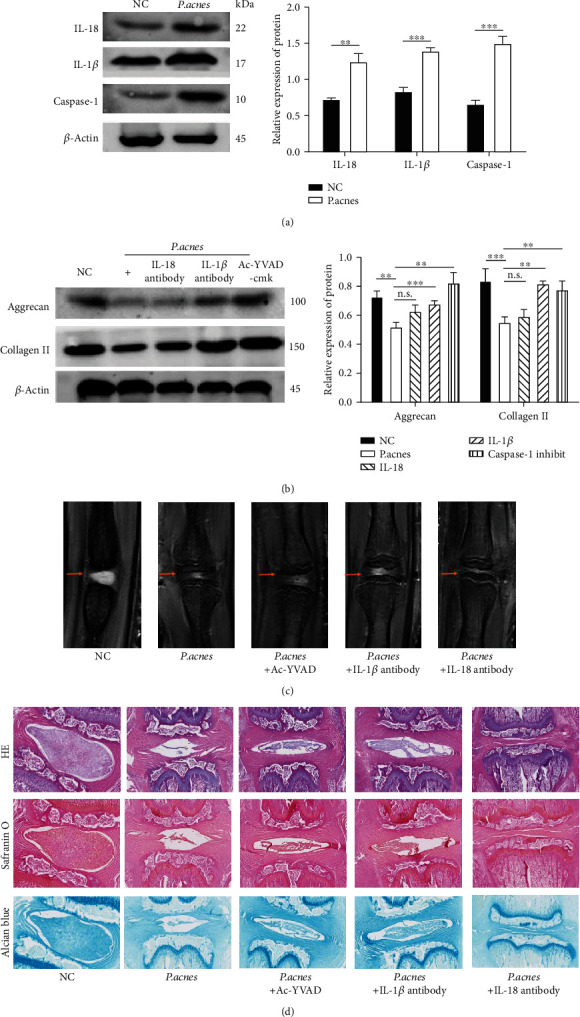
Pyroptosis of NPCs in *P*. *acnes*-inoculated IVDs of rats. (a) Inoculation of *P*. *acnes* into IVDs of rats resulted in an increase in cleaved IL-18, cleaved IL-1*β*, and cleaved caspase-1. (b) Inhibition of pyroptosis with Ac-YVAD-cmk or neutralization of pyroptotic products IL-1*β* or IL-18 ameliorated the degeneration of *P*. *acnes*-inoculated IVDs. (c) *P*. *acnes* inoculation induced remarkable degeneration of IVDs, indicated by the decrease in the area and signal darkening on MRI examination, while the damage caused by *P*. *acnes*-induced pyroptosis was reversed by the inhibition of caspase-1 with Ac-YVAD-cmk or neutralization of IL-1*β* or IL-18. (d) Histological analysis showed that inoculation of *P*. *acnes* into IVDs caused degeneration, which was apparent through the disappearance of the nucleus pulposus, disorganization of the annulus fibrosus, and infiltration of immune cells. In contrast, the inhibition of caspase-1 with Ac-YVAD-cmk or neutralization of IL-1*β* or IL-18 markedly ameliorated the degeneration of IVDs. One-way ANOVA and Bonferroni tests were used for statistical analysis. ^∗^*P* < 0.05, ^∗∗^*P* < 0.01, and ^∗∗∗^*P* < 0.001 when comparisons were made between groups. All of the values were expressed as mean ± SD.

To further identify the *P*. *acnes*-induced pyroptosis of NPCs in IVDs, we inoculated *P*. *acnes* into the IVDs of rats. As depicted in Figure 3(a), the expression of IL-1*β*, IL-18, and caspase-1 significantly increased, suggesting there was pyroptosis of NPCs in the nucleus pulposus. Furthermore, inoculation of *P*. *acnes* caused the downregulation of aggrecan and collagen II in IVDs, while inhibition of NPC pyroptosis with the caspase-1 inhibitor Ac-YVAD-cmk or neutralization of IL-1*β* or IL-18 with specific antibodies rescued the reduced aggrecan and collagen II (Figure 3(b)). On MRI examination, the IVD showed remarkable degeneration, which was reflected in the decreased area and darkening of the signal after *P*. *acnes* inoculation for 12 h. Furthermore, the damage caused by *P*. *acnes*-induced pyroptosis was reversed by the inhibition of caspase-1 with Ac-YVAD-cmk or neutralization of IL-1*β* or IL-18, as depicted in Figure 3(c). Histological analysis showed that inoculation of *P*. *acnes* into IVDs led to severe degeneration after 12 h, which was apparent through the disappearance of the nucleus pulposus, disorganization of the annulus fibrosus, and infiltration of immune cells. In contrast, the inhibition of caspase-1 with Ac-YVAD-cmk or neutralization of IL-1*β* or IL-18 markedly ameliorated the IVD degeneration, suggesting that NPC pyroptosis plays an important role in *P*. *acnes*-induced IVD degeneration (Figure 3(d)).

### 3.4. *P*. *acnes* Caused the Pyroptosis of NPCs via the ROS-NLRP3-Dependent Pathway

**Figure 4:**
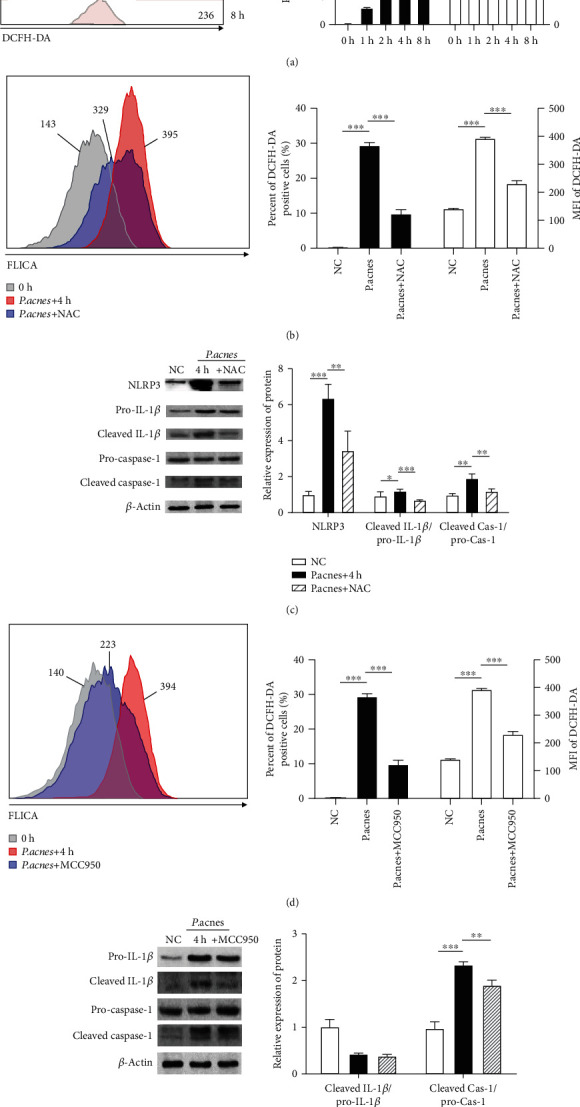
*P*. *acnes* caused the pyroptosis of NPCs via ROS-NLRP3 pathway. (a) Flow cytometry analysis suggested that *P*. *acnes* caused the overexpression of ROS in NPCs in a time-dependent manner. (b) Neutralization of *P*. *acnes*-induced ROS attenuated the pyroptosis of NPCs according to flow cytometry. (c) Neutralization of *P*. *acnes*-induced ROS attenuated the overexpression of pyroptosis-related proteins. (d) The pyroptosis of NPCs significantly decreased when NLPR3 was inhibited with MCC950, according to FCM analysis. (e) Inhibition of NLRP3 attenuated the overexpression of pyroptosis-related proteins. One-way ANOVA and Bonferroni tests were used for statistical analysis. ^∗^*P* < 0.05, ^∗∗^*P* < 0.01, and ^∗∗∗^*P* < 0.001 when comparisons were made between groups. All of the values were expressed as mean ± SD.

In our previous study, *P*. *acnes* had the ability to potently induce ROS in NPCs, and ROS have been proven to be key triggers for pyroptosis [17]. Therefore, we further explored whether *P*. *acnes* regulates the pyroptosis of NPCs via the ROS-NLRP3-dependent pathway. The flow cytometry results depicted in Figure 4(a) indicate that the level of ROS, which were stained with DCFH-DA, significantly increased after coculture with *P*. *acnes* in a time-dependent manner. Similarly, the pyroptotic proteins of NLRP3, the ratio of cleaved IL-1*β* to pro-IL-1*β*, and the ratio of cleaved caspase-1 to pro-caspase-1 significantly increased when NPCs were cocultured with *P*. *acnes* for 4 h. However, as shown in Figure 4(b), the percentage of pyroptotic NPCs significantly decreased when ROS were inhibited by NAC, suggesting that ROS are key factors in the initiation of NPC pyroptosis. In addition, the downstream pyroptotic proteins of NLRP3 significantly decreased when ROS were inhibited by NAC (Figure 4(c)), indicating that ROS regulate the *P*. *acnes*-induced pyroptosis of NPCs via NLRP3. Furthermore, as Figures 4(d) and 4(e) show, the inhibition of NLRP3 by MCC950 significantly reduced the number of pyroptotic NPCs and the expression of regulated pyroptotic proteins, suggesting that NLRP3 is a key protein in the regulation of *P*. *acnes*-induced pyroptosis of NPCs. Thus, we concluded that *P*. *acnes* induced the pyroptosis of NPCs via the ROS-NLRP3 signaling pathway.

## 4. Discussion

We previously reported that *P*. *acnes*, a low-virulence anaerobic bacterium that latently resides in IVDs, induced the pyroptosis of NPCs. The IL-1*β* and IL-18 released from pyroptotic NPCs led to a cascading degeneration effect on the surrounding normal NPCs, accelerating IVD degeneration. Finally, the ROS-NLRP3 signaling pathway played a critical role in the *P*. *acnes*-induced pyroptosis of NPCs.

Although it is a subtype of programmed cell death, pyroptosis is believed to result in collateral damage to surrounding cells or the extracellular matrix (ECM) and, therefore, differs from “harmless” apoptosis [18]. Caspase-1 is a key factor in the mediation of the classical signaling pathway of pyroptosis. Once activated by pathogens, cleaved caspase-1 assembles into the inflammasome body and converts the pro-forms of the inflammatory cytokines IL-1*β* and IL-18 into their active forms, resulting in cell apoptosis but with the concurrent release of inflammatory cytokines into the surrounding environment [18]. Additionally, the mature caspase-1 cleaves gasdermin D, which leads to the final pyroptotic cell death with the formation of plasma membrane pores [19].

*P*. *acnes* is one of the pathogens that induces the pyroptosis of cells. In studies on acne, *P*. *acnes* was able to induce the pyroptosis of human sebocytes or monocytes via the NLRP3 signaling pathway [14, 20]. Moreover, *P*. *acnes*-induced inflammasomes are thought to be one of the most important pathophysiological processes for acne or SAPHO syndromes [21, 22]. In the current study, we ascertained that *P*. *acnes* is capable of inducing the pyroptosis of NPCs, which was clear from the increase in pyroptotic proteins, upregulation of IL-1*β* and IL-18, and increase in pyroptotic cells in the flow cytometry results. More importantly, in the transwell study, neutralization of the pyroptotic products reduced the adverse effects on normal NPCs *in vitro* and prevented IVD degeneration *in vivo*, further indicating that *P*. *acnes*-induced pyroptotic NPCs are deleterious to the whole IVD. Recently, a study also verified the involvement of *P*. *acnes*-induced pyroptosis in disc degeneration, further confirming our findings [23].

The main destructive effect of pyroptotic cells depends on the release of the inflammatory factors IL-1*β* and IL-18. A wealth of studies have shown that IL-1*β* is a critical factor in the induction or acceleration of IVD degeneration [24]. On the one hand, increased IL-1*β* promotes the expression of a series of catabolic enzymes, such as ADAMTS or MMPs, destroying the ECM of IVDs [24]. On the other hand, IL-1*β* affects the homeostatic activities of IVD cells by inducing cell senescence and apoptosis [25]. Finally, IL-1*β* also triggers the infiltration of immune cells and induces an inflammatory cascade. All of these processes deteriorate the health of IVDs and, thus, lead to severe IVD degeneration. In this study, the neutralization of IL-1*β* released from pyroptotic NPCs significantly ameliorated IVD degeneration in *in vivo* and *in vitro* experiments, suggesting that IL-1*β* may be a key mediator in pyroptosis-induced IVD degeneration.

Unlike IL-1*β*, the role of IL-18 in IVD degeneration has rarely been studied. *In vitro*, IL-18 plays a catabolic role in IVDs, upregulating catabolic regulators and downregulating anabolic regulators [26]. In addition, the levels of serum IL-18 in patients are closely associated with the degree of IVD degeneration [26]. Furthermore, variations in IL-18 receptor genes may affect the risk of severe IVD degeneration and associated lower back pain [27, 28]. In this study, the neutralization of IL-18 also had an anticatabolic effect, but the effect was weaker than that of IL-1*β*, suggesting that IL-1*β* plays the main role in mediating pyroptosis-induced IVD degeneration.

Many endogenous and exogenous pathogens trigger cellular pyroptosis. Our previous study demonstrated that *P*. *acnes* upregulated the expression of ROS in NPCs via NADPH oxidase [17]. Moreover, inducing ROS is one of the ways endogenous pathogens trigger pyroptosis in cells [29, 30]. In this study, coculture with *P*. *acnes* increased ROS in a time-dependent manner in the NPCs, while the inhibition of ROS with NAC significantly reduced the expression of pyroptotic proteins and the proportion of pyroptotic NPCs in IVDs. In addition, NLRP3, a NOD-like receptor, which can sense intracellular pathogens, played a crucial role in *P*. *acnes*-induced pyroptosis, inducing the downregulation of pyroptotic proteins and the decrease in the proportion of pyroptotic NPCs. More importantly, NLRP3 expression was inhibited when ROS were inhibited by NAC, indicating that NLRP3 is a key downstream factor participating in the ROS-regulated pyroptosis of NPCs.

It should be mentioned that there were several limitations to this study. Some papers reported that *P*. *acnes* was able to directly activate NLRP3 in sebocytes [14]. Therefore, further study is needed to investigate whether *P*. *acnes* is capable of inducing pyroptosis of NPCs alone. In addition, the detailed molecular biological mechanisms of some critical pyroptotic proteins, such as NLRP3, ASC, and GSDMD, need to be further investigated in *P*. *acnes*-induced pyroptosis. Furthermore, whether the inflammatory products of *P*. *acnes*-induced pyroptosis would induce other IVD-related diseases, like the cytokine-induced neutrophil chemoattractant-1 (CINC-1) that induced lower back pain as we reported in the previous study, was an interesting topic for investigation in the future [31]. Finally, the issue that antigens, such as the antigens in bacterial walls or secreted metabolites, may play primary roles in mediating *P*. *acnes*-induced pyroptosis needs to be further investigated.

In conclusion, *P*. *acnes* induces the pyroptosis of NPCs via the ROS-NLRP3 signaling pathway, and pyroptotic NPCs cause an IVD degeneration cascade. Thus, targeting the *P*. *acnes*-induced pyroptosis of NPCs could be an alternative treatment strategy for IVD degeneration in the future.

## Data Availability

The datasets used and analyzed during the current study are available from the corresponding authors on reasonable request.

## Abbreviations

*P*. *acnes*: *Propionibacterium acnes*
IVD: Intervertebral disc
NPCs: Nucleus pulposus cells
MOI: Multiplicity of infection
GSDMD: Gasdermin D
FCM: Flow cytometry
NLRP3: NOD-like receptor 3
IL-1*β*: Interleukin-1 beta
IL-18: Interleukin-18
ROS: Reactive oxygen species.
